# Draft Genome Sequences of Type Strain *Sediminibacterium salmoneum* NJ-44 and *Sediminibacterium* sp. Strain C3, a Novel Strain Isolated from Activated Sludge

**DOI:** 10.1128/genomeA.01073-13

**Published:** 2014-01-16

**Authors:** Joaquín M. Ayarza, Eva L. M. Figuerola, Leonardo Erijman

**Affiliations:** a Instituto de Investigaciones en Ingeniería Genética y Biología Molecular Dr. Héctor N. Torres (INGEBI- CONICET), Buenos Aires, Argentina; b Departamento de Fisiología, Biología Celular y Molecular, Facultad de Ciencias Exactas y Naturales, Universidad de Buenos Aires, Buenos Aires, Argentina

## Abstract

The genus *Sediminibacterium* comprises species present in diverse natural and engineered environments. Here, we report for the first time the genome sequences of the type strain *Sediminibacterium salmoneum* NJ-44 (NBRC 103935) and *Sediminibacterium* sp. strain C3 (BNM541), isolated from activated sludge, a valuable model for the study of substrate-dependent autoaggregation.

## GENOME ANNOUNCEMENT

The genus *Sediminibacterium* is phylogenetically related to *Terrimonas* and *Niabella*, within the phylum *Bacteroidetes*. It was first described in 2008 by Qu and Yuan (1), who isolated the type strain *Sediminibacterium salmoneum* NJ-44 from a eutrophic reservoir in Beijing, China. Culture-independent methods allowed the detection of members of this genus in a variety of natural and engineered habitats (2–7).

*Sediminibacterium* sp. strain C3 was isolated from activated sludge treating wastewater from a petroleum refinery and deposited in the National Bank of Microorganisms (WDCM938) of the Facultad de Agronomia, Universidad de Buenos Aires, Argentina. Planktonic outgrowth of *Sediminibacterium* sp. C3 occurred after activated sludge deflocculated in response to chemical and thermal shocks. Remarkably, the isolate also showed substrate-dependent autoaggregation in pure culture (J. M. Ayarza, M. A. Mazzella, and L. Erijman, submitted for publication).

The 1,484-nucleotide-long amplicon of the 16S rRNA gene from isolate C3 has 99% similarity to the sequence of *S. salmoneum*. This, and the fact that the C3 strain appears to share many phenotypic traits with *S. salmoneum* NJ-44^T^, prompted us to sequence the genomes of both strains. *S. salmoneum* NJ-44^T^ was obtained from the Biological Resource Center (NBRC) of the National Institute of Technology and Evaluation, Japan (NITE).

Genomic DNA was isolated using the Wizard Genomic DNA purification kit (Promega) and sequenced using the 454 GS FLX Titanium system (Roche Diagnostics, Branford, CT) with 8-kb paired-end libraries at the Instituto de Agrobiotecnología Rosario (INDEAR), Argentina. Whole-genome sequencing of *Sediminibacterium* sp. C3 produced a total of 162,749 reads with an average read length of 355.61 bp. *De novo* assembly was done using Celera Assembler version 6.1, yielding a total consensus of 3,219,312 bp (39.20% G+C content) distributed in one main scaffold of 3,216,888 bp and three minor scaffolds of 1,479, 489, and 456 bp in length. *S. salmoneum* NJ-44^T^ sequencing generated a total of 277,742 reads with an average read length of 407.22 bp, which were assembled using 454 Newbler version 2.8, resulting in one scaffold of 3,246,830 bp (38.58% G+C content).

The genome sequences were annotated automatically using the Department of Energy Joint Genome Institute (DOE-JGI) Microbial Annotation Pipeline (8) through assembled sequence data submissions in the Integrated Microbial Genomes Expert Review system (IMG-ER). In *S. salmoneum* NJ-44^T^, 3,018 genes were predicted, with 2,969 protein-encoding genes and 49 RNA genes. Of the 3,011 genes predicted in *Sediminibacterium* sp. C3, 2,958 were protein-encoding genes and 53 were RNA genes. A total of 2,615 protein-encoding genes were conserved in both strains.

*Sediminibacterium* sp. C3 shows an average nucleotide identity (ANI) (9) with the *S. salmoneum* type strain of only 90.04%*. In silico* DNA-DNA hybridization (DDH) estimated using the Genome-to-Genome Distance Calculator (GGDC 2.0) Web server (10) was 37.20% ± 2.48%, which has an associated probability of 0.97% that two strains belong to the same species. Therefore, both analyses suggest that C3 represents a novel species of the genus *Sediminibacterium*.

### Nucleotide sequence accession numbers.

The whole-genome shotgun projects for *Sediminibacterium* sp. C3 and *S. salmoneum* NJ-44^T^ were deposited at DDBJ/EMBL/GenBank under accession no. AXUM00000000 and AXZP00000000, respectively.
